
JOURNAL INFORMATION
==============================
NLM Title Abbreviation: ScientificWorldJournal
Journal ID: ScientificWorldJournal
NLM Journal ID: 101131163
Journal ID: TSWJ

The Scientific World Journal

ISSN: 2356-6140
EISSN: 1537-744X
Publisher: Wiley

ARTICLE INFORMATION
==============================
PMCID: PMC5848706
PMID: 18836664
DOI: 10.1100/tsw.2008.133
Article ID: 521042
Article version: 1
Subjects: Clinical Image

Benign Hepatic Portal Venous Gas in a Critically Ill Patient

Gorospe Emmanuel C. *
Department of Medicine, Johns Hopkins Bayview Medical Center, Johns Hopkins University School of Medicine, Baltimore, MD, USA
*Emmanuel C. Gorospe: egorosp1@jhmi.eduAcademic Editor: Daniel Batlle

Publication date: 2008
Electronic publication date: 2007 Oct 3
Volume: 8
First page: 951
Last page: 952
Received 2008 Mar 31; Revised 2008 Sep 14; Accepted 2008 Sep 28
Copyright: Copyright © 2008 Emmanuel C. Gorospe.
Copyright year: 2008
License: This is an open access article distributed under the Creative Commons Attribution License, which permits unrestricted use, distribution, and reproduction in any medium, provided the original work is properly cited.
License URL: https://creativecommons.org/licenses/by/3.0/

Keywords: hepatic portal venous gas, bowel ischemia, computed tomography

==============================
Clinical Image
*TheScientificWorldJOURNAL* (2008) 8, 951–952
ISSN 1537-744X; DOI 10.1100/tsw.2008.133

TheScientificWorldJOURNAL
www.thescientificworld.com

### Benign Hepatic Portal Venous Gas in a Critically Ill Patient

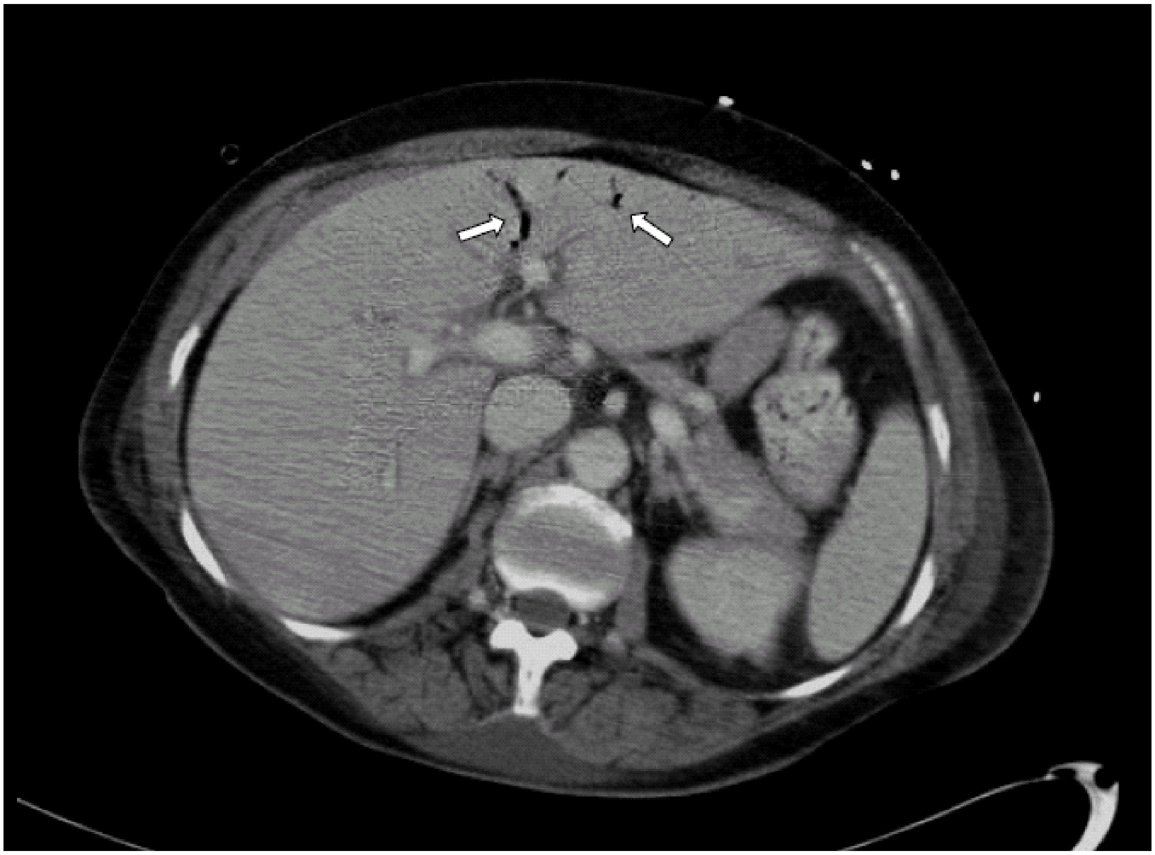

**FIGURE.** CT of the abdomen showing hepatic portal venous gas (see arrows).

Emmanuel C. Gorospe

*Department of Medicine, Johns Hopkins Bayview Medical Center, Johns Hopkins University School of Medicine, Baltimore, MD*

**E-mail**: egorosp1@jhmi.edu

*Received March 31, 2008; Revised September 14, 2008; Accepted September 28, 2008; Published October 3, 2007*

**KEYWORDS**: hepatic portal venous gas, bowel ischemia, computed tomography

©2008 with author.
Published by TheScientificWorld; www.thescientificworld.com

Hepatic portal venous gas (HPVG) formation is regarded as a sign of a serious intra-abdominal process due to its association with bowel necrosis[1], perforation[2], obstruction[3], and mesenteric infarction[4]. It is attributed to either disruption of intestinal mucosa, gas production in the hepatic portal system, or to iatrogenically increased intestinal lumen pressures such as in nasogastric intubation[4,5]. This abdominal CT scan is from a 62-year-old female who was admitted to the intensive care unit for hypercarbic respiratory failure from chronic obstructive pulmonary disease exacerbation. After 2 days of nasogastric tube placement, the patient's alkaline phosphatase increased from 84 to 210 U/L. She has no history of recent endoscopic gastrointestinal examination. Her abdominal exam was unremarkable. The rest of the chemistry panel, including lactic acid, was within normal limits except for an elevated gamma-glutamyl transpeptidase of 419 U/L. Blood cultures were negative. An abdominal CT scan revealed new HPVG formation as compared to an earlier scan on admission. There were no CT scan findings of free intra-abdominal gas, pneumatosis intestinalis, bowel or mesenteric ischemia, lymphadenopathy, gall bladder disease, pancreatitis, bowel obstruction, or inflammation. Given these findings, no aggressive intervention was done. On hospital discharge, her liver enzymes normalized and follow-up abdominal ultrasound showed resolution of HPVG. Understanding that there are multiple conditions that can precipitate the development of HPVG, its finding should prompt a thorough diagnostic evaluation to immediately rule out a catastrophic intra-abdominal condition. The patient's overall clinical condition should be placed into perspective when deciding on the need for any invasive intervention.

### REFERENCES

1. Gan, H.N., Tan, K.Y., Chong, C.K., and Tay, K.H. (2006) Finding hepatic portal venous gas in an adult patient: its significance. *Singapore Med. J.* **47,** 814–816.
2. Duggal, A., Rankin, R.N., and Wall, W.J. (2007) Hepatic portal venous gas from perforated sigmoid diverticulitis. *Can. J. Surg*. **50,** E19–20.
3. Hong, J.J., Gadaleta, D., Rossi, P., Esquivel, J., and Davis, J.M. (1997) Portal vein gas, a changing clinical entity. Report of 7 patients and review of the literature. *Arch. Surg*. **132,** 1071–1075.
4. Monneuse, O., Pilleul, F., Barth, X., Gruner, L., Allaouchiche, B., Valette, P.J., and Tissot, E. (2007) Portal venous gas detected on computed tomography in emergency situations: surgery is still necessary. *World J. Surg.* **31,** 1065–1071.
5. Yamamuro, M. and Ponsky, J.L. (2000) Hepatic portal venous gas: report of a case. *Surg. Today* **30,** 647–650.

**This article should be cited as follows:**

Gorospe, E.C. (2008) Benign hepatic portal venous gas in a critically ill patient. *TheScientificWorldJOURNAL* **8**, 951–952. DOI 10.1100/tsw.2008.133.
